# Regulatory Mechanism of Endothelin Receptor B in the Cerebral Arteries after Focal Cerebral Ischemia

**DOI:** 10.1371/journal.pone.0113624

**Published:** 2014-12-05

**Authors:** Anne-Sofie Grell, Rushani Thigarajah, Lars Edvinsson, Ajoy Kumar Samraj

**Affiliations:** 1 Department of Clinical Experimental Research, Glostrup research institute, University of Copenhagen, Glostrup, Denmark; 2 Division of Experimental Vascular Research, Department of Clinical Sciences, Solvegatan 17, Lund University, Lund, Sweden; National University of Singapore, Singapore

## Abstract

**Background and Purpose:**

Increased expression of endothelin receptor type B (ET_B_R), a vasoactive receptor, has recently been implied in the reduced cerebral blood flow and exacerbated neuronal damage after ischemia-reperfusion (I/R). The study explores the regulatory mechanisms of ET_B_R to identify drug targets to restore normal cerebral artery contractile function as part of successful neuroprotective therapy.

**Methods:**

We have employed *in vitro* methods on human and rat cerebral arteries to study the regulatory mechanisms and the efficacy of target selective inhibitor, Mithramycin A (MitA), to block the ET_B_R mediated contractile properties. Later, middle cerebral artery occluded (MCAO) rats were used to substantiate the observations. Quantative PCR, immunohistochemistry, western blot and wire myograph methods were employed to study the expression and contractile properties of cerebral arteries.

**Results:**

Increased expression of specificity protein (Sp1) was observed in human and rat cerebral arteries after organ culture, strongly correlating with the ET_B_R upregulation. Similar observations were made in MCAO rats. Treatment with MitA, a Sp1 specific inhibitor, significantly downregulated the ET_B_R mRNA and protein levels. It also significantly reduced the ET_B_R mediated cerebrovascular contractility. Detailed analysis indicated that ERK1/2 mediated phosphorylation of Sp1 might be essential for ET_B_R transcription.

**Conclusion:**

Transcription factor Sp1 regulates the ET_B_R mediated vasoconstriction in focal cerebral ischemia via MEK-ERK signaling, which is also conserved in humans. The results show that MitA can effectively be used to block ET_B_R mediated vasoconstriction as a supplement to an existing ischemic stroke therapy.

## Introduction

Ischemic stroke causes tissue infarction and malfunction of neural networks in the brain leaving patients with permanent disability and reduced cognitive sensory motor function [1]. Stroke is primarily a vascular disorder that adversely affects neurons [2]. In focal cerebral ischemia a larger part of brain tissue surrounding the ischemic core, the penumbra, is salvageable given that the cerebral blood circulation is promptly re-established [3]. Persistent ischemic cascades due to changes in shear stress following ischemia-reperfusion (I/R) have been found to increase certain contractile G-protein coupled receptors (GPCRs) in the cerebral arteries and the associated brain tissue [4]. This has been documented in humans after stoke as well as in experimental focal and global cerebral ischemia [4], [5]. In particular, the expression levels of contractile endothelin receptors type A and type B (ET_A_R and ET_B_R) are upregulated in cerebral vascular smooth muscle cells and are speculated to play a pivotal role in the physiological and pathological processes of the brain post-stroke. Upon endothelin-1 (ET-1) binding, the endothelin receptors increase the contractile function of the cerebral arteries thereby reducing the blood flow to the affected area of the brain. It results in exacerbated neuronal damage. ET-1 is one of the most potent vasoconstrictors that are shown to play a pivotal role in post-ischemic hypoperfusion. Increased plasma levels of ET-1 have been documented in several stroke patients and it has been shown to correlate with worst clinical outcome [6]. Unlike ET_A_R, which has been implied in exacerbated neuronal damage [7], [8], the role of ET_B_R is not clearly established. However, compelling numbers of evidences indicate that ET_B_R might play an important role in post-ischemic hypoperfusion associated neuronal damage [4]. Extensive research in this field has so far not yielded any clinically effective neuroprotective agent; hence there is a need for the identification of new compounds for stroke therapeutics. Re-establishing cerebral blood flow is a crucial part of stroke therapeutics. Therefore we asked whether interfering with ET_B_R-mediated vasoconstrictive properties to reestablish cerebral blood flow will help neurons to recover from post ischemic-hypoperfusion. In order to answer the question, understanding of the regulatory mechanism of ET_B_R becomes essential. Hence the study is designed to identify agent(s) that could specifically inhibit ischemia-induced smooth muscle ET_B_R expression without affecting the normal contractile properties of cerebral arteries. In this study, using *in vitro* and *in vivo* experimental methods, we show that Sp1 regulates the smooth muscle specific expression of ET_B_R in the cerebral arteries of rodents and humans. Phosphorylation of Sp1 by ERK1/2 might be crucial for the DNA binding to initiate ET_B_R transcription. In addition, we show that MitA efficiently blocks the ET_B_R upregulation to restore normal vascular contractile function after ischemia-reperfusion.

## Materials and Methods

### Animal handling and ethics

All experiments were conducted in full compliance with the guidelines set forth in the European Council's Convention for the Protection of Vertebrate Animals Used for Experimental and other Scientific Purposes. The experimental procedures used in the study are approved by the Lund University Animal Ethics Committee (M43-07). Regional Ethical Review Board in Lund, Sweden (LU-818-01) approved the experiments on human cerebral arteries. The study conforms to the principles outlined in the Declaration of Helsinki and subjects gave informed written consent. Male Sprague-Dawley rats weighing 300-350 g were obtained from Taconic, the Netherlands, and kept in standard housing conditions.

### Chemicals and Reagents

Dulbecco's modified Eagle's medium (DMEM) contained L-glutamine (584 mg/L) supplemented with penicillin (100 U/ml) and streptomycin (100 µg/ml) (Gibco BRL, Paisley, UK). The inhibitors MitA and U0126 were purchased from Tocris and Sigma and dissolved in phosphate buffered saline (PBS), 1 mM, and dimethylsulfoxide (DMSO), 10 mM, respectively. All general chemicals that are used in the experiments were purchased from Sigma.

### Transient Middle Cerebral Artery Occlusion (MCAO) and Mithramycin A treatment

Transient MCAO was induced in male Sprague-Dawley rats by inserting a filament intraluminally as described by Memezawa et al. [9]. Anesthesia was induced by 4.5% isoflurane in N_2_O∶O_2_ (70∶30). Thereafter the animals were kept anesthetized by inhalation of 1–1.5% isoflurane in N_2_O/O_2_. To confirm proper occlusion, a laser-Doppler probe (Perimed, Järfälla, Sweden) was fixed on the skull (1 mm posterior to the bregma and 6 mm from the midline on the right side) to measure the regional cortical blood flow. Physiological parameters were measured before the occlusion and controlled within physiological limits. The body temperature was maintained by a rectal temperature probe connected to a homeothermic blanket set to maintain the body temperature at 37°C. An occlusion is ensured when there is a sharp drop (>70%) in the cerebral blood flow as visualized by laser Doppler. Two hours after MCAO, in order to establish reperfusion and normalization of blood flow, the filament was withdrawn by briefly anaesthetizing the rats. At desired time points post-MCAO rats were anesthetized and sacrificed by decapitation followed by isolation of middle cerebral arteries (MCAs) from the brain. The left MCA that was not subjected to cerebral ischemia served as control. Rats that did not show sharp drop in cerebral blood flow were excluded from the study. For the treatment studies, MCAO rats were divided into two groups, one group received intraperitoneal injection of MitA (250 µg/kg in 300 µl) immediately after reperfusion and a second dose after 24 hours, while the other group received vehicle, PBS.

### *Ex vivo* culturing of intact cerebral arteries

*Ex vivo* culturing of intact cerebral arteries were performed as described in detail previously [10]. Segments (3–4 mm long, diameter 200–300 µm) were incubated in serum-free Dulbecco's Modified Eagle's Medium with or without inhibitors for desired time points in a humidified CO_2_ incubator. Similar methods were employed to culture human cerebral arteries obtained from four patients aged 55, 68, 51 and 22 while undergoing neurological surgery.

### RNA extraction and quantitative real-time PCR

RNA extraction and cDNA synthesis was performed according to the kit manufacturers protocols respectively (Machery-Nagel, Düren, Germany and Qiagen, Hilden, Germany). Quantitative real time PCR was performed using SYBR Green kit (Qiagen) on a CFX384 Real-Time System (Bio-rad) and normalized to the housekeeping genes β-actin, GAPDH and EF1-α. All primers were obtained from SAbiosciences (Qiagen).

### Immunohistochemistry

Immunohistochemistry was performed as described previously [10]. 4% paraformaldehyde fixed MCAs were embedded in Tissue-TEK (Sakura Finetek Denmark ApS, Copenhagen, Denmark) and sectioned into 10 µm thick cryosections (Microm HM500 M; Thermo Scientific, Walldorf, Germany). On each microscope slide three sections were collected (Menzel-Gläzer, Braunschweig, Germany). Mounted sections were permeabilized in phosphate buffer solution (PBS) containing 0.25% Triton X-100 for 15 min and blocked 1 hour in blocking buffer containing PBS, 0.25% TritonX-100, 1% bovine serum albumin (BSA) and 5% normal donkey serum, and then incubated with the following primary antibodies overnight at 4°C: Sheep anti-ET_B_R, 1∶250, (Enzo life sciences, ALX-210-506A-C250), rabbit anti-Sp1, 1∶250, (Millipore, 07-645), rabbit anti-phopho-Sp1(T739), 1∶250 (Sigma, SAB4300670), goat anti-SM22, 1∶500 (Abcam, ab10135). Sections were subsequently incubated 1 hour at room temperature in dark with respective secondary antibodies conjugated with fluorochromes (Jackson ImmunoResearch, Europe Ltd., Suffolk, UK) diluted 1∶250 in PBS containing 0.25% Triton X-100, 1% BSA and 3% normal donkey serum. The sections were washed with PBS, mounted with anti-fading Vectashield mounting medium (Vector Laboratories Inc., Burlingame, CA, USA) and visualized using an epifluorescence microscope (Nikon 80i; Tokyo, Japan). Similar procedure was carried out for the negative controls except the primary antibody step.

### Western blots

Western blot analysis was carried out as described previously [11]. Cerebral arteries were lysed by sonication on ice in lysis buffer (20 mM Tris/HCl, pH 7.4, 1% Triton X-100, 10% glycerol, 150 mM NaCl, 1 mM PMSF, and 1 µg/ml each leupeptin, antipain, chymostatin, and pepstatin A) and centrifuged (15min, 14,000×*g*). For Western blot analysis 15–20 µg protein was separated by a 4–12% gradient SDS-PAGE (RunBlue, Expedeon, Cambridgeshire, UK) and transferred to a polyvinylidene difluoride membrane (Amersham Bioscience, Freiburg, Germany). The membrane was blocked with 5% BSA in Tris-buffered saline (TBS)/0.2% Tween for 1 hour at room temperature and incubated overnight with the primary antibodies at 4°C. The following antibodies were used in the experiments: Anti-sheep ET_B_R (Enzo Life Sciences, ALX-210-506A-C250), 1∶1000, anti-rabbit Sp1 (Millipore, 07-645), 1∶1000, phospho-Sp1, T453 (Abcam, ab59257), 1∶1000, anti-rabbit ERK1/2 (Cell Signaling Technology, Danvers, MA, USA, #9102), 1∶2000, anti-rabbit phospho-ERK1/2 (Cell Signaling Technology, #9101), 1∶1000, anti-mouse Actin (Abcam, ab11003), 1∶1000, and anti-goat β-Actin (Abcam, ab8229), 1∶1000. Membranes were washed four times with TBS/0.02% Triton X-100 and incubated with the respective HRP-conjugated secondary antibody for 1 hour at room temperature (Pierce/Thermo Scientific, Rockford, IL, USA). After extensive washing, the proteins were visualized by enhanced chemiluminescent staining using ECL reagents (Amersham Bioscience).

### *In vitro* pharmacology

Contractile responses of rat cerebral arteries were measured by recording isometric tension in a Mulvany Halpern myograph (Danish Myo Technology A/S, Aarhus, Denmark). Vessel segments were mounted in a temperature-controlled tissue bath (37°C) using two stainless steel wires (40 µm), then stretched to 90% of the normal internal circumference, which is equivalent to a relaxed vessel that is under a transmural pressure of 100 mmHg. Contraction induced by 60 mM K^+^ was considered as reference for maximal contractile capacity of the vessel. Endothelial function was evaluated by inducing contraction by 5-hydroxytryptamine (5-HT, 0.3 µmol) followed by addition of carbachol (10 µmol) to relax the vessel. Concentration curves for various contractile receptors were obtained by cumulative addition of agonists: Sarafotoxin 6c (S6c) was used as selective endothelin receptor B agonist (10^−14^ to 3·10^−7^ mol/L) and ET-1, an endothelin receptor A and B agonist, was used in the presence of BQ788 which is a selective ET_B_ receptor inhibitor (10^−14^ to 3·10^−7^ mol/L).

## Results

### Focal cerebral ischemia induces increased expression of Sp1 and ET_B_R in rat cerebral arteries

**Figure 1:**
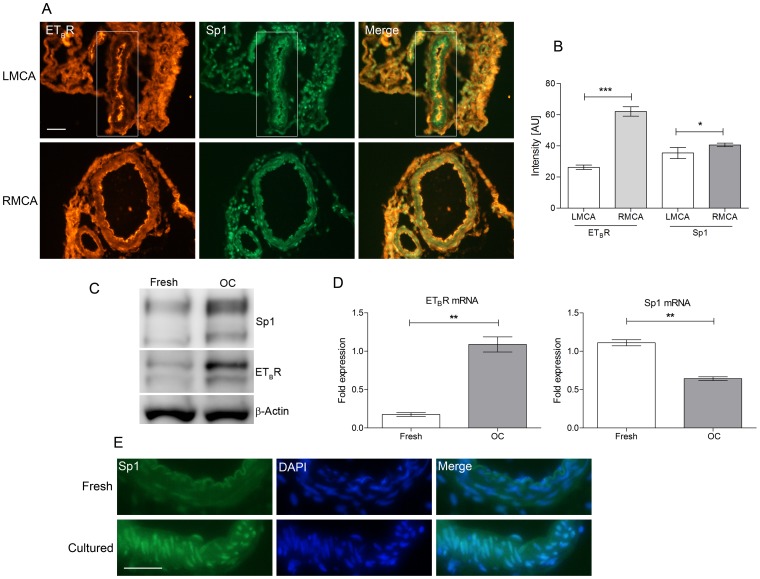
Increased vascular smooth muscle expression of Sp1 and ET_B_R after MCAO and organ culture. **A.** Representative immunohistochemical stainings of MCA sections from MCAO rats after 48 hrs. of I/R (LMCA: non-occluded side and RMCA: occluded side) (n = 4 per group). Scale bar is 50 µm. **B.** Bar graphs show the statistical significance of intensity measurements of Sp1 and ET_B_R in figure 1A (***P<0.001, *P<0.05). **C.** Representative western blot shows protein levels of ET_B_R and Sp1 in cultured cerebral arteries at 24 hrs. (n = 4 per group). **D.** Quantitative PCR analysis of ET_B_R and Sp1 mRNA levels in cultured cerebral arteries at 6 hrs. (n = 4 per group, **P<0.01). **E.** Immunohistochemical staining showing the expression and localization of Sp1 in fresh and 24 hrs. cultured MCAs (n = 4 per group). Scale bar is 50 µm. **Statistics**: Values are presented as mean ± S.E.M. Mann-Whitney test was performed between two groups for statistical significance.

MCAO was performed for 120 minutes followed by 48 hours of reperfusion. Immunohistochemical analysis was carried out to evaluate the expression of Sp1 and ET_B_R in the right MCA (RMCA, occluded side) and left MCA (LMCA, non-occluded side). We noted an increased Sp1 immunoreactivity, primarily in vascular smooth muscle cells of the MCA on the occluded side, correlating with the increased expression of ET_B_R (Fig. 1A). Fluorescence intensity measurements of the sections showed a significant increase in the expression of both proteins (Fig. 1B). An *in vitro* method, where the cerebral arteries were cultured in serum-free medium for a defined period of time, was employed to further study the regulatory mechanism of the receptor. The method is shown to mimic several of the vascular changes during focal cerebral ischemia [12]. This method is in general used for detailed analysis of molecular alterations in the vessel walls. Similar to the observations in cerebral arteries after MCAO, western blot analysis showed a notable increase in the expression of Sp1 and ET_B_R in cerebral arteries cultured for 24 hours (Fig. 1C). We noted two reactive bands in the blots for Sp1 and ET_B_R, indicating that the proteins might have undergone post-translational modification or are expressed in their respective isoforms [13], [14]. Quantitative PCR analysis after 6 hours culturing showed a significant increase in the ET_B_R mRNA and an inverse correlation between Sp1 mRNA and protein was observed (P<0.01, Fig. 1D). With immunohistochemistry we noted increased immunoreactivity for Sp1 in the smooth muscle cell layer of cultured MCAs supporting the western blot observation (Fig. 1E and fig. S1). Sp1 was primarily localized in the nucleus of the vascular smooth muscle cells. Such changes in the Sp1 protein expression without increased levels of mRNA indicate that Sp1 is probably regulated by cap-independent translation mechanisms [15].

### Inhibition of transcription factor Sp1 downregulates ET_B_R expression in rat and human cerebral arteries

**Figure 2:**
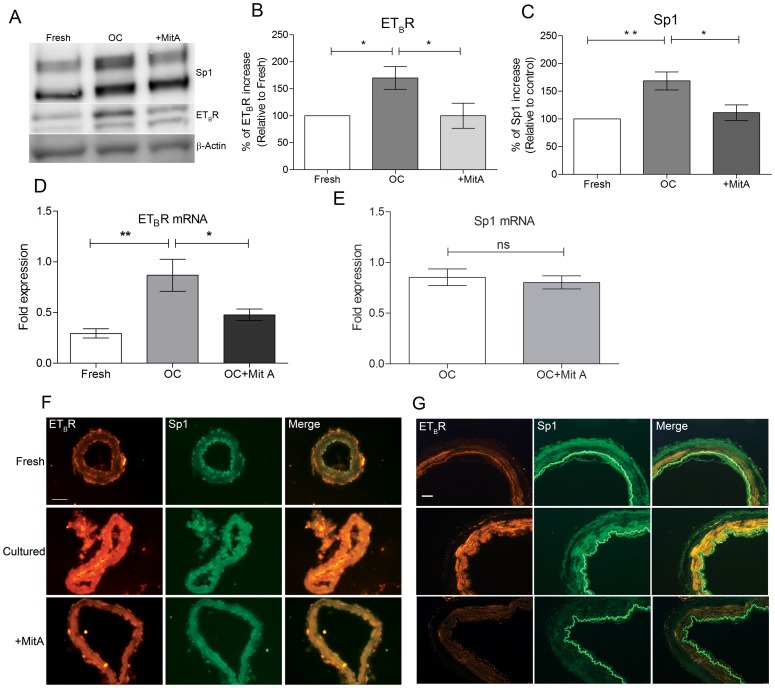
Inhibition of transcription factor Sp1 blocks ET_B_R upregulation *ex vivo* in rat and human cerebral arteries. **A.** Representative western blot for Sp1 and ET_B_R protein levels in cultured cerebral arteries with and without 5 µM MitA at 24 hrs. **B and C.** Bar graphs show the statistical significance of protein expression and inhibition of Sp1 and ET_B_R in figure 2A (Fresh n = 7, OC n = 7, MitA n = 6, **P<0.01, *P<0.05). **D and E.** Quantitative PCR analysis of ET_B_R and Sp1 mRNA levels in fresh and 24 hrs. cultured MCA segments with and without MitA treatment (n = 6 per group, **P<0.01, *P<0.05). **F.** Representative immunohistochemical stainings of MCAs cultured in the presence or absence of MitA at 24 hrs. (n = 4 per group). Scale bar is 50 µm. **G.** Immunohistochemical stainings of cultured human cerebral arteries show ET_B_R and Sp1 immunoreactivity with and without MitA treatment at 24 hrs. (n = 4 per group). Scale bar is 50 µm. **Statistics**: Values are presented as mean ± S.E.M. One-way ANOVA and Dunnett's multiple comparison test was done for figure B, C and D while Mann-Whitney test was done for figure E for statistical significance.

DNA foot printing studies have shown that Sp1 competitively binds to GC rich motifs of various promoters [16]. MitA has been shown to selectively displace Sp1 from its binding site to inhibit the transcriptional activity [17],[18]. Therefore, the effect of Sp1 inhibition on ET_B_R upregulation was investigated by culturing cerebral arteries in the presence or absence of MitA. Western blot analysis showed a notable reduction in the ET_B_R expression of cerebral arteries cultured with MitA (5 µM). Among the two reactive bands for ET_B_R, expression of one band was notably reduced indicating a possible isoform-specific inhibition by MitA. Similar trend was observed in the expression pattern of Sp1 in the presence of MitA (Fig. 2A). Intensity measurements of the protein expression levels of Sp1 and ET_B_R are significantly altered in the presence or absence of MitA after organ culture (P<0.05) (Fig. 2B and C). Quantitative PCR analysis showed a significant reduction in the ET_B_R mRNA upon treatment (P<0.05) while the expression of Sp1 mRNA remained unaffected (Fig. 2D and E). In conjunction, immunohistochemical analysis showed a marked reduction in ET_B_R expression in MCAs incubated with MitA (Fig. 2F). No significant reduction in the expression of Sp1 was observed as MitA is expected to competitively displace Sp1 from the DNA not the protein expression. However, when compared to MCAs cultured in absence of the inhibitor, MitA-treated arteries showed a diffused Sp1 staining pattern, which probably is due to its displacement and subsequent degradation of the protein (Fig. 2F).

Since the human ET_B_R promoter is shown to contain a unique binding site for Sp1 proximal to the transcriptional start site, we evaluated the relevance of our findings in human cerebral arteries. Due to the difficulties in obtaining stroke specimens, the organ culture method was chosen to validate the findings in normal human cerebral arteries. Cerebral arteries were cultured in the presence or absence of MitA for 24 hours. Supporting the rodent *in vivo* and *in vitro* data, a prominent increase in the ET_B_R expression was found correlating with the nuclear expression of Sp1 (Fig. 2G). In the presence of MitA, we observed a notable reduction in the smooth muscle cell specific expression of ET_B_R and the change in the Sp1 expression was less or not significant. These results are in line with the observations made in the rat MCAs. To demonstrate that Sp1 and ET_B_R expression is in the smooth muscle cells of MCAs, we stained human cerebral arteries with smooth muscle action 22 (SM22), a specific marker for muscle cells (See figure S1). Taken together figures 2G and S1 give clear indications that Sp1 and ET_B_R are expressed in the SMCs of MCA.

### Phosphorylation of Sp1 by ERK1/2 is pivotal for the ET_B_R transcription

**Figure 3:**
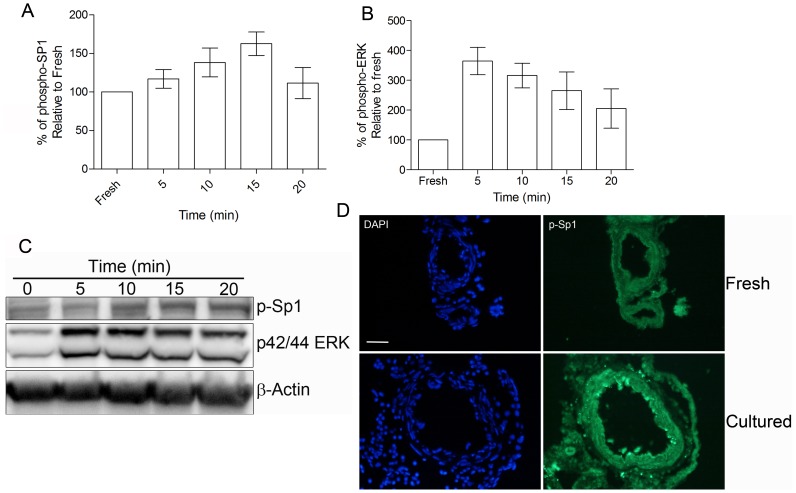
ERK1/2 mediated phosphorylation of Sp1 is essential for ET_B_R upregulation. **A.** and **B.** Bar graphs show the time-dependent changes in the phosphorylation status of Sp1 (T453) and ERK1/2 based on the intensity measurements of western blots (n = 4 per group). No statistical difference between groups was observed. **C.** Representative western blot showing the time-dependent changes in the phosphorylation status of Sp1 (T453) and ERK1/2 in culture conditions. **D.** Representative immunohistochemical staining of MCA sections showing the phosphorylation status of Sp1 (T739) after 120 min of culturing (n = 4 per group). **Statistics**: Values are presented as mean ± S.E.M. One-way ANOVA and Bonferronis multiple comparison test was performed for statistical significance.

Transcription factor Sp1 mediates signal transduction either by multiple protein interaction or by posttranslational modification such as phosphorylation [19]. Studies have demonstrated that ERK1/2 directly phosphorylates Sp1 on threonine 453 (T453) and 739 (T739) *in vitro* and *in vivo* [20]. Hence, the status of phosphorylated Sp1 (p-Sp1) and ERK1/2 (p-ERK1/2) was evaluated at various time points in culture conditions. Western blot analysis showed that vascular ERK1/2 is activated as early as 5 min after initiating the incubation. When the blots were probed for phosphorylated Sp1 at T453, a known ERK1/2 target, we found that Sp1 could be phosphorylated within 5 minutes of ERK1/2 activation (Fig. 3A, B and C). Intensity measurements of those blots showed time dependent increase of p-Sp1 levels correlating with the increased p-ERK1/2 but not statistically significant (Fig. 3A and B). To substantiate the phosphorylated status of Sp1, sections of organ cultured MCAs were immunohistochemically analyzed for phospho-T739, another ERK1/2 target. It showed an increased immunoreactivity for phospho-T739. These observations suggest that the phosphorylation of T453 and T739 could be essential for the nuclear translocation and DNA binding function of Sp1 (Fig. 3D). Even though the intensity measurements of immunoblots indicated that p-Sp1 levels slowly decrease over time, we still observed sustained basal level of p-Sp1 up to six hours in culturing conditions (data not shown). The observation of ERK1/2 activation is in line with the previous studies indicating that MEK-ERK signaling is essential for ET_B_R upregulation [10], [21]. Taken together these observations suggest that ERK1/2 mediated phosphorylation of Sp1 could be essential for the ET_B_R transcription.

### Sp1 transcription factor regulates ET_B_R expression and vascular function in rat MCAs

**Figure 4:**
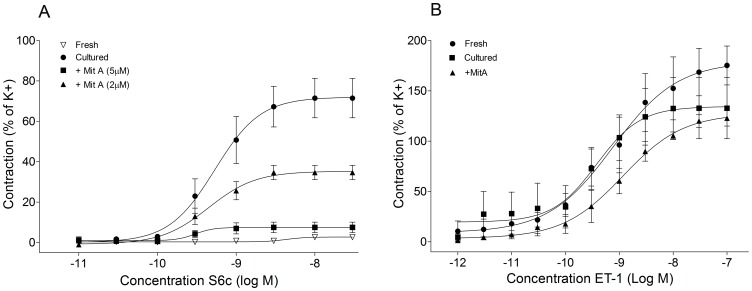
Inhibition of Sp1 blocks ET_B_R mediated cerebrovascular contractility *ex vivo*. **A.** Graphs depict concentration-response curves of 24 hrs. organ cultured (cultured) and non-cultured (Fresh) MCA segments elicited by cumulative application of S6c (ET_B_R specific) in the presence or absence of MitA in a dose dependent manner (Fresh n = 6, OC n = 5, 2 µM MitA n = 3, 5 µM MitA n = 4). OC vs. 2 µM MitA; not significant. OC vs. 5 µM MitA; P = 0.0004. **B.** Graphs show concentration-response curves of cultured and fresh MCA segments elicited by cumulative application of ET-1 in the presence or absence of MitA (Fresh n = 5, OC n = 4, 5 µM MitA n = 5). No significant difference between the groups was observed. **Statistics:** Values are presented as mean ± S.E.M. One-way ANOVA and Dunnett's multiple comparison test was performed for statistical significance.

Next we asked whether the inhibition of Sp1 with MitA could restore normal vascular contractile function by preventing the increase in ET_B_R-mediated vasoconstriction after *in vivo*; stroke and *in vitro*; organ culture. Changes in the contractile properties of MCA were investigated using an ET_B_R specific agonist, S6c, in a wire myograph system. The MCAs were cultured with and without varying concentrations of MitA for 24 hours in serum-free conditions. MCAs treated with MitA showed significant reduction in the contractile responses to S6c compared to vehicle-treated MCAs (Fig. 4A). A 2 µM concentration of the inhibitor was sufficient to bring noticeable reduction in ET_B_R-mediated contraction. A 5 µM concentration was sufficient to bring the S6c contraction to the basal level of contraction as evidenced in fresh MCAs (P<0.05, Fig 4A). Meanwhile, up to 10 µM of MitA, contractile responses mediated by ET_A_R remained unaltered suggesting that Sp1 specifically interferes with the ET_B_R regulation (Fig 4B).

**Figure 5:**
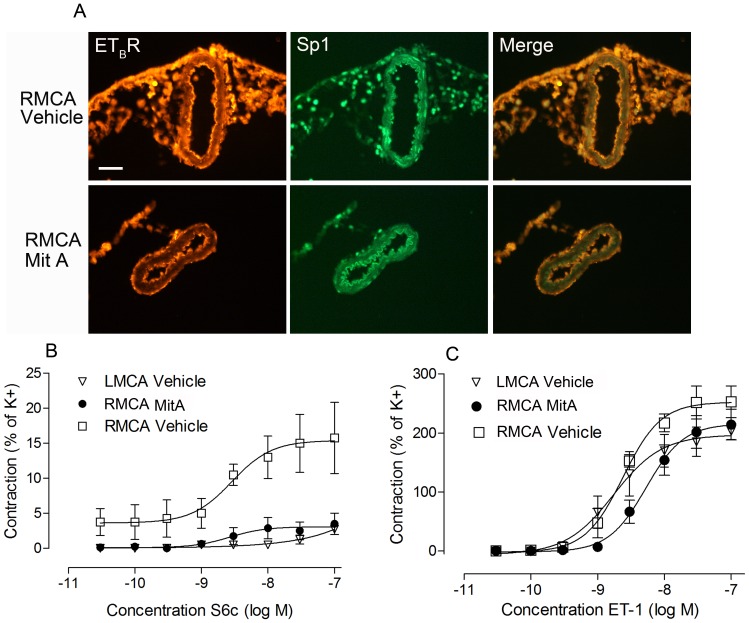
Sp1 inhibition downregulates ET_B_R expression and vascular contractility after MCAO. **A.** Representative ET_B_R and Sp1 immunostainings in the MCA on the occluded side (RMCA). Stainings show changes in the expression levels after treatment with MitA compared to vehicle (n = 4 per group). **B.** Graphs depict concentration-response curves of the MCA segments elicited by cumulative application of S6c (ET_B_R specific) of MitA and vehicle treated rats after MCAO. Vehicle vs. RMCA MitA, P<0.0001 and LMCA Vehicle vs. RMCA MitA, not significant. **C.** Graphs depict concentration-response curves of the MCA segments elicited by cumulative application of ET-1 of MitA and vehicle treated rats after MCAO. No significant difference between the groups (Vehicle, n = 4 and MitA, n = 6). **Statistics:** Values are presented as mean ± S.E.M. One-way ANOVA and Dunnet's multiple comparison test was performed to obtain statistical significance.

The findings from MitA inhibition and Sp1-regulated ET_B_R vasoconstriction of rat MCAs *in vitro* were then validated in experimental stroke. A profound decrease in the vascular smooth muscle ET_B_R receptor expression was observed after treatment with MitA (Fig. 5A). Furthermore, MitA treatment significantly prevented the ischemia-induced ET_B_R mediated vasoconstriction compared to vehicle treated rats (P<0.001, Fig 5B). In addition, confirming the *in vitro* data, MitA treatment did not significantly alter the contractile function mediated by the ET_A_R in MitA treated rats compared to vehicle treated rats indicating that MitA might be specifically interfering with the ET_B_R transcription mechanism (Fig. 5C). Taken together these results indicate that MitA could serve as a potential pharmacological agent to block ET_B_R mediated cerebrovascular contraction after I/R.

## Discussion

Stroke is a vascular disease; hence functionally intact cerebral arteries are essential criteria for any kind of successful neuroprotective therapy. Cerebrovascular receptors play a pivotal role in keeping the vascular function intact. These receptors are deregulated upon ischemic insult to the brain and contribute to the occurrence of persistent cascade of ischemic events after I/R that results in exacerbated neurological damage [22], [23]. Recent studies have demonstrated that the smooth muscle cell specific expression of ET_B_R might play a pivotal role in I/R mediated constriction of cerebral arteries [10], [24]. In this study we documented a significant increase in the Sp1 (transcription factor) expression in the vascular smooth muscle cells of MCAs, coinciding with increased expression of ET_B_R after I/R (Fig. 1A and B). Sp1 is a zinc finger-containing DNA binding protein, which can activate or repress transcription upon DNA binding. Previous studies have shown that inhibition of Sp1 with MitA is neuroprotective [25], [26]. Based on the correlation between the Sp1 mRNA and protein expression we suggest that the Sp1 expression is regulated by a probable “cap independent translation mechanism” where translation occurs with the existing mRNA through an internal ribosomal entry site (Fig 1C, D and E). The Sp1 mRNA has been shown to contain such internal ribosomal entry sites [15]. In addition based on our observations, we speculate that Sp1 might undergo post-translational modification to regulate the gene expression. Such modifications might be essential for its DNA binding, protein stability and the ability of Sp1 to interact with other proteins as indicated by previous studies [14], [20], [27]. In this context direct phosphorylation of Sp1 at T453 and T739 by ERK1/2 *in vitro* and *in vivo* has been shown to be pivotal for its transcriptional activity [20]. Our *in vitro* studies showed that both target amino acids are phosphorylated by ERK1/2 indicating that Sp1 is a downstream target of ERK1/2 (Fig 3). This observation is in agreement with previous studies showing ERK1/2 inhibition blocking ET_B_R upregulation [10], [21]. Even though the post-translational modification (phosphorylation) is not statistically significant, we strongly believe such smaller changes might be sufficient to induce significant changes in gene expression pattern contributing to the changes in phenotype.

As a next step we employed Sp1 inhibitor MitA, to test the role of Sp1 on ET_B_R transcription *in vitro*. Treatment with MitA resulted in down regulation of ET_B_R expression and restoration of contractile properties that are similar to non-cultured MCAs (Fig. 2 and 4A). Most notably, the inhibitor did not alter responses mediated by ET_A_R or the total contractile response to high potassium buffer as well as high concentrations of MitA (10 µM, data not shown), indicating that MitA is target specific (Fig 4B). This observation is in agreement with the reports suggesting that MitA does not affect global protein synthesis with a special emphasizing on the specificity of the agent [26], [28]. As a next step the study was extended to human cerebral arteries to test the clinical relevance of the findings. When human cerebral arteries were treated with MitA, in culture conditions, we observed a significant reduction in the ET_B_R expression. As substantiation, the ET_B_R mediated contractile properties were validated in MCAO rats after MitA treatment. A significant reduction in the ET_B_R mediated cerebrovascular contractility was evidenced in the treated MCAO rats. Taken together our study indicates that ERK1/2 and Sp1 could serve as potential pharmacological targets in aiding focal cerebral ischemia treatment. Preclinical studies with MEK inhibitor U0126 have indicated that the inhibitor is the most effective when treated immediately after I/R [29]. Due to the robustness of ERK1/2 activation, the U0126 becomes ineffective if treated few hours after I/R. Since Sp1 is acting further downstream of ERK1/2, it becomes an attractive target for the treatment of ET_B_R mediated vasoconstriction and associated complications. However, in order to establish MitA as a potential agent, a long-term preclinical study is essential to document the impact of the agent on the inflammatory cytokines, improvement in stroke volume and positive neurological outcome. A detailed toxicology report of MitA is readily available and it has been used in humans to treat number of malignancies [30],[31]. Therefore, given that MitA has a significant positive impact on the above-mentioned parameters, the compound can quickly be available for clinical trials.

In conclusion, this study has unraveled the regulatory mechanism involved in the augmented expression of ET_B_R after cerebral ischemia. We demonstrate that the cerebrovascular specific increase of ET_B_R is regulated by the transcription factor, Sp1 and suggest that ERK1/2 mediated phosphorylation of Sp1 could be essential for the initiation of ET_B_R transcription.

## Supporting Information

Figure S1 **Sp1 is expressed in the smooth muscle cells of cerebral arteries.** Cultured human cerebral arteries were co-stained for the expression of Sp1 (Green) and smooth muscle cell specific marker a-SM22 (Orange). Staining indicate that the Sp1 protein is expressed in the smooth muscle cells.
(TIF)
