# Supplementary figures and images for: Regulatory Mechanism of Endothelin Receptor B in the Cerebral Arteries after Focal Cerebral Ischemia
Source: PLoS One. 2014 Dec 5;9(12):e113624. doi: 10.1371/journal.pone.0113624 (PMC4257531; doi:10.1371/journal.pone.0113624)

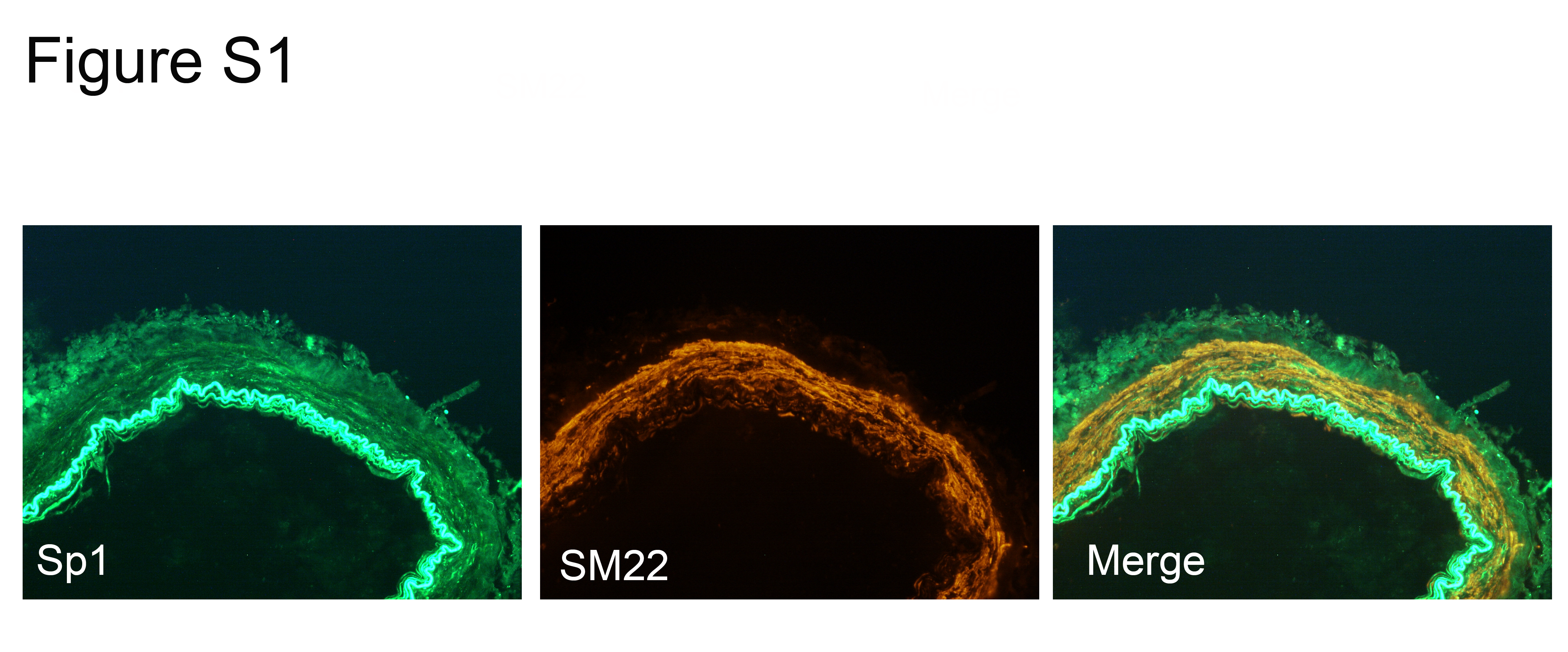

Supplement: Figure S1 — Sp1 is expressed in the smooth muscle cells of cerebral arteries. Cultured human cerebral arteries were co-stained for the expression of Sp1 (Green) and smooth muscle cell specific marker a-SM22 (Orange). Staining indicate that the Sp1 protein is expressed in the smooth muscle cells. (TIF) [file pone.0113624.s001.tif]
